# Comparison of postoperative pain after root canal treatment using reciprocating instruments based on operator’s experience: A prospective clinical study

**DOI:** 10.4317/jced.54037

**Published:** 2017-07-01

**Authors:** Marc García-Font, Fernando Duran-Sindreu, Carmen Calvo, Juan Basilio, Fransesc Abella, Akram Ali, Miguel Roig, Juan-Gonzalo Olivieri

**Affiliations:** 1 Department of Restorative Dentistry and Endodontics, Universitat Internacional de Catalunya, Barcelona, Spain; 2 Department of Integrated Dentistry, Universitat Internacional de Catalunya, Barcelona, Spain

## Abstract

**Background:**

The aim of the present study was to compare clinically the incidence of postoperative pain after endodontic treatment using the Reciproc System, taking into account the operator’s experience.

**Material and Methods:**

One hundred patients scheduled for routine endodontic treatment were enrolled in this study. Endodontic treatment was carried out in a single visit by undergraduate and postgraduate students. The chemomechanical preparation of root canals was performed with Reciproc instruments. Pretreatment and postoperative pain was recorded using a visual analogue scale (VAS). Postoperative pain and the need for analgesic consumption were assessed at 4, 8, 16, 24, 48 and 72 hours post-treatment. The data were analyzed using the Mann–Whitney U and Chi-Square test, and the significance was set at *P*<0.05.

**Results:**

The mean value of pain after root canal treatment was 1.13±1.94 and 1.91±2.07 on a VAS between 0 and 10 in treatments performed by undergraduate and postgraduate students, respectively. There was a significant difference in the incidence of postoperative pain between the two groups (*P*<0.05).

**Conclusions:**

The prevalence of postoperative pain was high in the treatments performed by postgraduate students in comparison with undergraduate students. This suggests that operator experience has an influence on the prevalence of postoperative pain after root canal treatment.

** Key words:**Post-endodontic pain, root canal treatment, reciprocating systems, Expert operators Inexperienced operators.

## Introduction

Root canal treatment is a common procedure that aims to preserve the tooth by treating diseases of the dental pulp and periradicular region (1). Therefore, the teaching of endodontics warrants an important place in the curriculum of any dental training school. Undergraduate endodontic teaching has made significant headway in educational approaches to knowledge, techniques, and materials. These advances have improved the ability of dental students to diagnose and treat pulpal and periradicular diseases (2).

Postoperative pain, described as the perception of any annoyance after root canal treatment, is reported by 25-40% of patients, regardless of their pulp and periradicular status (3-5). Post-endodontic pain usually occurs during the first 2 days after treatment, and generally diminishes after a few hours (6-8). However, it sometimes persists for several days (9-11). According to a recent systematic review (9), the prevalence of pain during the first 24 hours after root canal treatment is 40%, falling to 11% after 7 days. Thus, pain control, both during and after root canal treatment, poses a huge challenge to the clinician (12).

Post-endodontic pain can be caused by several factors (9). The most important seems to be related to the instrumentation procedure, which can provoke an acute periapical inflammatory response secondary to mechanical, chemical and/or microbial injury to the periradicular tissues (13). Inflammation may be produced by the extrusion of dentinal debris, pulp tissue, microorganisms, and irrigants to the periapical tissues during chemomechanical preparation (14). The intensity of pain seems to be correlated with the extent of tissue damage (15).

In order to simplify endodontic instrumentation and improve the fracture resistance of rotary nickel-titanium (NiTi) files, the concept of shaping canals with a single-file was introduced in endodontics (16). According to Yared *et al.* (16), reciprocating movement is preferable to continuous rotation to reduce the risk of instrument fracture and deformation of the root canal. Mandel *et al.* (17) reported that the incidence of instrument separation decreases with experience, indicating the need to improve operator competence through learning and experience. Therefore, the concept of using a single-file reciprocating instrument to prepare the entire root canal by inexperienced operators is interesting as it considerably reduces the learning curve as a result of technique simplification for root canal preparation (18). Reciproc instruments (VDW, Munich, Germany) can shape canals with minimal preparation. Reciproc instruments are characterized by an ‘‘S’’-shaped cross-section with a gradually decreasing taper after the apical 3 mm and spiral flutes with high cutting efficiency.

Post-endodontic pain has been investigated in several studies (13,19-21); however, there is little knowledge about the incidence of postoperative pain after treatment with reciprocating instruments. Moreover, to our knowledge, no studies have evaluated the relationship between operator experience and postoperative pain after root canal treatment using a reciprocating system. Thus, the purpose of this prospective clinical study was to compare the incidence of postoperative pain after root canal treatment performed by undergraduate and postgraduate students using the Reciproc system.

## Material and Methods

This prospective clinical study was conducted in patients who attended or were referred for routine endodontic treatment at the University Dental Clinic (Universitat Internacional de Catalunya, Sant Cugat del Vallés, Barcelona, Spain). The study was reviewed and approved by the Institutional Ethics in Research Committee.

-Patient selection

A total of 100 consecutive adults were included in this study. The sample size calculation, based on an error of alpha of 0.05 and a power of 80%, indicated that a sample size of 38 in each group would be required to detect differences. Hence, 50 teeth assigned to each group were considered sufficient to ensure a representative sample.

The aims and design of the study were explained to the patients, who gave their oral and written informed consent. Prior to treatment, the medical and dental history of the patients was taken. Gender, age, tooth type and location in the arch, as well as preoperative pain intensity, periapical condition, and pulp diagnosis (vital or necrosis) were all recorded. The exclusion criteria included: immunosuppressed patients, pregnancy, consumption of any type of medication before treatment, history of intolerance to nonsteroidal anti-inflammatory drugs, patients under 18 years old, patients with pacemakers, root canal retreatment, the presence of internal or external resorption teeth, and open apex.

-Treatment Protocol

Of the total sample of 100 patients, 50 were treated by undergraduate students and 50 by students of the endodontic master postgraduate program. The treatment protocol was the same for both groups. After clinical examination, the cold test (Endo-Frost; Coltene-Whaledent, Langenau, Germany) was used to determine pulp vitality, which was verified by the presence or absence of bleeding from the root canals during endodontic access preparation. If there was no response after 5 seconds of applying a cotton pellet and non–bleeding, the teeth were classified as necrotic. The treatment was performed on all the patients in a single visit.

Anesthesia was performed with local infiltration using 4% articaine with 1:100.000 epinephrine (Ultracain, Normon, Madrid, Spain). Rubber dam isolation was placed after access cavity preparation with sterile round diamond burs and Endo-Z burs (Dentsply Maillefer, Ballaigues, Switzerland). Working length (WL) was established with a #10 K-file and an apex locator (RootZX; J. Morita, Tokyo, Japan), and confirmed with a periapical radiograph.

Root canal shaping was performed in accordance with the manufacturer’s instructions. For each canal, the Reciproc instrument (VDW, Munich, Germany) selection was as follows: the R25 files (25.08) were used in narrow and curved canals when the #20 K-type file (Dentsply) could not achieve the WL passively. For medium and large canals in which the #20 or #30 K-type files were placed passively up to the working length, the R40 (40.06) or R50 (50.05) files were used, respectively. Then, 1 mL of 4.2% sodium hypochlorite (NaOCl) was placed in the access cavity before introducing the Reciproc instrument into the canals, in a slow in-and-out pecking motion, without completely removing the file from the root canal, and the range of motion did not exceed 3-4 mm. Debris on the instrument was removed using alcohol-soaked gauze, after every 3 in-and-out (pecking) motions. During instrumentation, the canals were irrigated with 4.2% NaOCl solution with a plastic syringe and a closed-end needle (Max-I-probe; Kerr-Hawe, Bioggio, Switzerland). Apical patency was maintained throughout the shaping procedure by using a #10 K type file between each in-and-out pecking motion. The instruments were driven in a torque control endodontic motor (VDW Silver/Gold Reciproc motor, VDW GmbH) in Reciproc All Mode. Each tooth was shaped with a single-use file, after which the files were discarded.

Upon completion the instrumentation procedure, 1ml 10% citric acid was used to remove smear layer. A final flush with 4.2% NaOCl was performed, and the canals were dried with paper points. The canals in both groups were filled with a warm gutta-percha obturation technique. AH-Plus cement (Dentsply, DeTrey GmbH, Konstanz, Germany) was used as the root canal sea-ler. Each canal access was sealed with a flowable composite (Tetric; IvoclarVivadent AG, SchaanFurstentum, Liechtenstein) and the access opening was temporarily filled with a Cavit restoration (ESPE dental, Seefeld, Germany).

-Assessment of Postoperative Pain and Statistical Analysis

Pre- and postoperative pain was assessed using the Huskinsson (22) visual analogue scale (VAS). According to the values recorded on the VAS, the pain was classified as no pain (0), slight pain (0.1-3.9), moderate pain (4-6.9), or severe pain (7-10), as described in a previous study (19). None of the patients was prescribed medication after treatment. The recommended medication for pain control, if required, was ibuprofen 600 mg every 8-12 h.

According to our previous study (19), postoperative pain was assessed in two ways: the highest value of pain recorded during the first 72 h post-treatment, and the patient’s need to take analgesics. The Mann–Whitney U and chi-square tests were used for statistical analysis using Statgraphics Centurion XV software version 15.2.06 (SPSS Inc., Chicago, IL). Significance was set at *P* < .05.

## Results

 Table 1 outlines the baseline demographic and clinical features of the study groups. As mentioned, the variables (sex, tooth type, position of tooth in the arch, presence or absence of symptoms before treatment, and pulpal status) may influence postoperative pain. To rule out the influence of these variables as confounding factors in the results of this study, a statistical test was first conducted to confirm that there was no difference in terms of the variables mentioned above between undergraduate and postgraduate groups (*P* > .05).

**Table 1:**
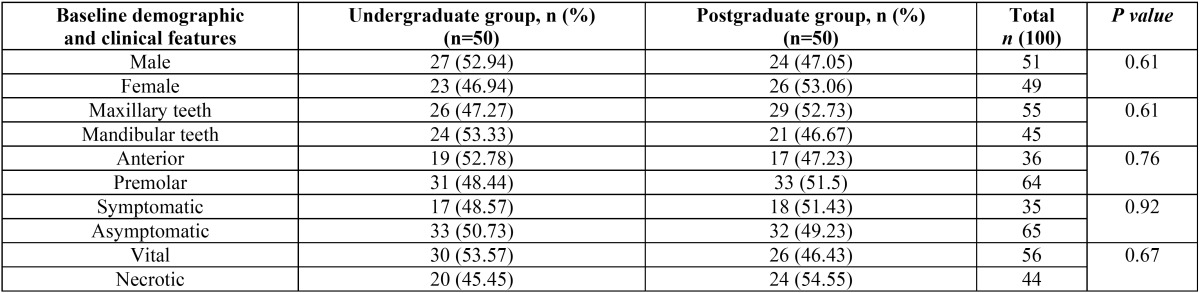
Baseline demographic and clinical features in the study groups.

The mean age of the patients in the undergraduate group was 45.3 years, and in the postgraduate group was 46.1 years. The Mann–Whitney test showed no significant difference in age distribution between the two groups (*P* > .05).

-Pretreatment pain, Post-treatment pain and intake of analgesics

The mean baseline pretreatment pain in the undergraduate student group and the Endodontic Postgraduate student group was 1.99±3.20 and 2.35±2.75, respectively, with no significant differences (*P* > .05).

Overall, the mean value of pain after root canal treatment performed by undergraduate students was 1.13±1.94 on a VAS scale between 0 and 10, while in treatments performed by postgraduate students, the mean value of postoperative pain experienced by patients was 1.91±2.07. There was a significant difference in the incidence of postoperative pain between the two groups (*P*< .05).

**Figure 1:**
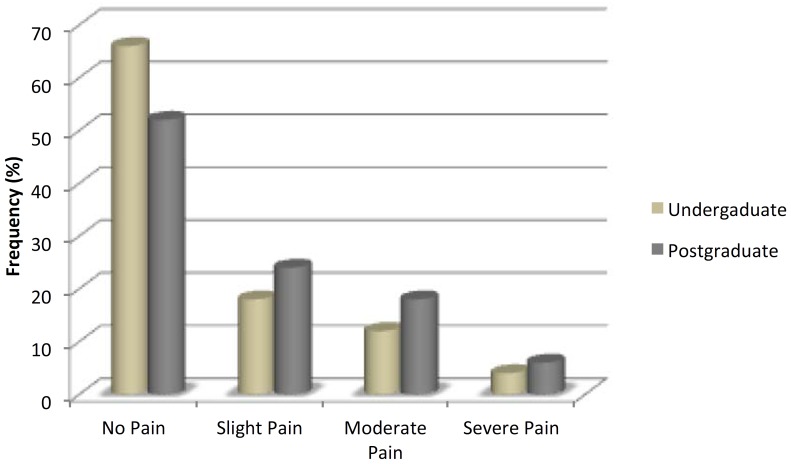
Severity of postoperative pain according to VAS.

The mean incidence of postoperative pain was 41% (41/100). Slight, moderate, and severe pain was experienced by 21%, 15% and 5% of the patients, respectively. In the undergraduate group 18%, 12%, and 4%, of patients experienced slight, moderate, and severe pain, respectively, whereas in the postgraduate group, 26 patients (52%) reported no pain, 12 (24%) slight pain, 9 (18%) moderate pain, and only 3 (6%) reported severe pain (Fig. 1). The evolution of the intensity of preoperative pain showed that, in both groups, patients who had a higher intensity of preoperative pain had a higher incidence of postoperative pain (*P* < .05).

Of the 100 patients, only 30 (30%) reported having taken analgesics. In the undergraduate group, 28% of patients (14/50) reported having taken analgesics, a value similar to that obtained in the postgraduate group (16 out of 50 patients; 32%), showing no significant difference (*P* > .05). The consumption of analgesics in both groups was significantly higher in patients who experienced more pain (*P* < .001).

## Discussion

The aim of this clinical study was to assess and compare the incidence, intensity, and duration of postoperative pain after root canal treatment with Reciproc instruments, taking into account the operator’s experience.

We found that patients treated by postgraduate students had a significantly higher prevalence of postoperative pain than those treated by undergraduate students (*P* = 0.01). These results are in accordance with Walton *et al.* (23), who reported significantly lower post-obturation pain between patients treated by undergraduate operators and patients treated by residents or faculty members.

Although there is no obvious explanation for the difference in the results, we could hypothesize that postgraduate students performed more difficult treatments. However, patient distribution in this study was not related according to difficulty, and the two groups treated the same types of teeth. Some authors (19,21,24) reported that sex, tooth type, position of the tooth in the arch, presence or absence of symptoms before treatment, and pulpal status could be factors associated with an increased risk of pain after root canal treatment. The statistical analysis showed that these factors were not confounding factors in the postoperative pain recorded between the two groups (*P* > 0.05). We found no differences in sex, tooth type, pulpal and periapical status, position of the tooth in the arch, and presence or absence of symptoms before treatment between the two groups. Another possible explanation may be that, in the present study, the undergraduate students took considerably longer to perform root canal procedures. This could result in a longer exposure of tissues to irrigant solutions, which could drastically reduce bacteria (23).

However, our results differ from those obtained by Wong *et al.* (25) and Glennon *et al.* (26), who reported that operator experience had no influence on the incidence of postoperative pain. The variance of our results could be due to differences in operator experience. In the two studies mentioned, the operators had some experience in root canal treatment, which was in contrast to our study, in which the operators were still studying an endodontic masters program.

Although significant differences were found between the prevalence of postoperative pain between the two groups (*P* = 0.01), the mean pain in both groups was less than 2. According to several authors (19,27-29), a postoperative pain below 2 is considered slight/mild, and which has been defined as a weak discomfort that did not require analgesics and does not influence everyday activities.

Despite differences in post-treatment pain results, only 2 and 3 patients in the undergraduate and postgraduate group, respectively, experienced severe pain, described as pain unrelieved by analgesics, forcing the patient to give up his/her daily activities and make an emergency appointment with a clinician.

Postoperative pain after root canal treatment is a multivariable problem in daily clinical practice. However, the most important variable seems to be inflammation caused by the debris/bacterial extrusion into the periapical tissues (9,10,12,13).

The results of *in vitro* studies demonstrate that all instrumentation systems produce debris/bacterial extrusion beyond the foramen (30-32). However, there is no consensus in the literature that reciprocating systems produce a greater amount of debris extrusion in the apical region than rotary systems, which could be related to postoperative pain. Bürklein and Schäfer (30) reported an increased amount of debris extrusion with reciprocating instruments than with rotary techniques. On the other hand, other authors (31-33) have found that the use of reciprocating systems does not imply a greater apical debris extrusion.

Postoperative pain after root canal treatment with reciprocating instruments is not associated with increased pain compared with preparations performed by conventional full-sequence rotary systems (5,34). Cruz Junior *et al.* (28) showed that the apical extrusion with the Reciproc system was not clinically significant.

In our study, we shaped the canals with copious irrigation to minimize debris/bacterial extrusion. The instrument was advanced to the WL with minimal apical pressure. After every 3 in-and-out (pecking) movements, the Reciproc instrument was cleaned and the debris removed from the instrument until WL was achieved. The WL was determined with an EAL and confirmed radiographically.

We did not consider the apical diameter as a variable to evaluate postoperative pain. The Reciproc file was chosen based on the manufacturer’s recommendations. It can be argued that apical enlargement may have an impact on postoperative pain; nevertheless, a recent study on postoperative pain after apical enlargement demonstrated that patients with and without apical enlargement experienced the same level of postoperative pain and the same need for analgesic intake (35).

Postendodontic pain is highly subjective and is influenced by several factors (9). Pain management before, during and after treatment should be an integral part of dental treatment. Because the measurement of subjective variables is such a huge challenge, different scales and methods have been used to assess postoperative pain. In the current study, the Huskinsson VAS (22), a continuous scale on which all intermediate values are recorded, was used. It is easily understood by patients, and is a simple, valid and reliable scale that has been widely used in previous endodontic research (21,27,36,37). In a present study we used a 10 cm line because Revill *et al.* (38) in their study showed that it is significantly more accurate than a 5 cm line (21,27,38).

We used a Reciproc system, which offers easier root canal preparations and requires a simpler learning curve (39). This concept is especially appropriate for undergraduate students with no experience in endodontics.

One of the main concerns in pain research is the subjective evaluation of pain. Thus, any decision to take analgesics will depend on this subjectivity (35). In our study, the need to take analgesics was used to measure postoperative pain. Both groups showed similar values regarding the intake of analgesics (28% of the patients in undergraduate group and 32% in the postgraduate group). These results are in accordance with those obtained by Ali *et al.* (19), who reported that 28.5% of patients need analgesics after root canal treatment. Our findings lead us to conclude that the need for analgesics suggests a link to high levels (moderate or intense) of postoperative pain.

Within the limitations of this study, we conclude that operator experience has an influence on the prevalence of postoperative pain after root canal treatment. Our findings showed that the Reciproc System is a good option for operators without experience in endodontics.
